# Genome reports: enhancing rigor, reproducibility, and relevance

**DOI:** 10.1093/g3journal/jkad249

**Published:** 2023-11-23

**Authors:** Noah K Whiteman

**Affiliations:** Department of Integrative Biology, University of California, Berkeley, 3040 Valley Life Sciences Building, Berkeley, CA 94720, USA; Department of Molecular & Cell Biology, University of California, Berkeley, 3040 Valley Life Sciences Building, Berkeley, CA 94720, USA

## Abstract

In this Editorial, Senior Editor for Genome Reports Noah Whiteman discusses G3’s new approach to the article type, outlining the specialized peer review process used. In addition to a traditional, open-ended review, Genome Reports receive a structured review that evaluates the article for best practice in both sequencing strategy and data analysis, reproducibility, and data availability. This carefully designed approach to Genome Reports supports G3’s commitment to serving the community through rapid, robust, and helpful peer review.

As the Senior Editor for Genome Reports at G3: Genes|Genomes|Genetics, I write to share some exciting news and to request that you spread the word. G3's Genome Reports are the Genetics Society of America's primary venue for publishing peer-reviewed manuscripts that share high-quality genome assemblies, descriptions of diversity panels, and related genome analyses for eukaryotic organisms. We welcome submissions for important cell lines and collections/populations for understanding genetic diversity in all organisms.

G3 was born from a commitment to serve our community. The journal was started to provide a forum for the rapid dissemination of high-quality, foundational research—particularly research that generates useful genetic and genomic information. During a Fall 2022 Board of Senior Editors meeting discussions centered around how the latest wave of technology has inspired our community to sequence and characterize the genomes of a diverse array of organisms at a rapidly increasing pace. This year alone we published reports on organisms such as boll weevil (*Anthonomus grandis)*, rusty patched bumble bee (*Bombus affinis*), gyrfalcon (*Falco rusticolus*), threespot damselfish (*Dascyllus trimaculatus*), Kentucky bluegrass (*Poa pratensis*), longnose gar (*Lepisosteus osseus*), and butternut (*Juglans cinerea*). These are just a few of the many excellent reports that can be found at G3.

In response to this increase in accessibility of genome sequencing and to support the publication of these reports, we have developed a specific peer review process for Genome Reports. Here, we explain how the process works at G3. First, we assembled a terrific front-line team of Associate Editors, led by me as Senior Editor with the support of Editor-in-Chief Lauren McIntyre and Deputy Editor Rob Kulathinal. We recruited Ricardo Mallarino at Princeton University, Polly Campbell at UC-Riverside, Joe Parker at CalTech, and Kevin Vogel at University of Georgia who joined editors Pär K. Ingvarsson, Andrew Whitehead, Arun Sethuraman, Antonis Rokas, Esther Betran, J.J. Emerson, Gustavo de los Campos, Jeffrey Ross-Ibarra, and many others to develop our editorial process. A total of 28 Associate Editors have helped guide more than 80 submissions through peer review with more than 70 Genome Reports published this year!

Our goal was to ensure that manuscripts were reviewed for reproducibility and usefulness and that this review was fair, balanced, and streamlined. To meet this goal, we beta-tested several strategies and thank the community for their support during this process. We have now implemented a structured peer review of the methods for reproducibility to compliment the more traditional open-ended peer review.

What this means in practice is that 1 of the 2 peer reviewers recruited for each Genome Report is asked to specifically evaluate whether the manuscript meets stringent criteria for best practices in sequencing strategy and data analysis; that the approaches used are reproducible; and that the public has access to the primary data, assembled genomes, and relevant repeat masks and variant calls.

We developed these methods review criteria in collaboration with the editorial team and with input from our community of authors and reviewers.

Because one of our goals as members of the genetics and genomics research community is to be transparent in how our peer review process works, we would like to share the template we ask peer reviewers to use for genome report methods and include it below.

We hope that by providing this information, the community will be able to prepare their manuscripts in a way that is likely to meet all of these criteria from methodological, reproducibility, and biological relevance perspectives.

Please consider sending your manuscripts that report novel genome assembly resources to G3. We will continue to strive for fast, courteous, and helpful feedback enabling robust peer review coupled with rapid publication to support your efforts to understand genomes from organisms across the tree of life.

## Methods review

Genomes are a valuable and useful resource for the community when they are representative and well assembled. We are specifically interested in your opinion about how well the current manuscript describes the important details of this genome assembly including:

- Does the report provide a stand-alone assembly or have they integrated their data with a previous assembly? If the latter, are all the data that are being integrated into the new data well described, referenced, and accessible?
- How were the individual(s) selected/sampled and are the sample characteristics well described (e.g. sex, tissue, genotype). Is the sampling strategy employed going to result in a useful reference for the species or will the usefulness be limited by the choices made in sampling?
- Is there sufficient attention to detail and quality control? Have the authors reported on the quality and scale of the raw data? Is the size of the assembly reasonable compared with the known/expected genome size and are the reported summary statistics (e.g. N50, L50, NG50, NGA50, percentage of Ns) acceptable?
- Has the genome been annotated for protein-coding genes, transposable elements (TEs), and other repetitive DNA and has this process been described in enough detail to reproduce the annotation? Do the results indicate the genome is likely to contain most of the conserved proteins (e.g. reasonable BUSCO score)?
- Are the bioinformatics used for assembly using up-to-date and appropriate tools? And is the approach described in detail and accompanied by scripts?
- If there are “other” analyses (e.g. structural variation, repetitive elements, TE's), are these described in detail and accompanied by scripts?

We would also appreciate your comments on whether the results presented in the manuscript:

- provide a useful resource for the target community,
- include a rationale for the genome(s) for the general readership of the journal, and
- are clear and easy to read for a nonspecialist.

